# Adolescent Hip Pain Due to Iliopsoas Abscess

**DOI:** 10.7759/cureus.71761

**Published:** 2024-10-18

**Authors:** Rita Lages Pereira, Sara Nogueira Machado, Liliana Macedo, Jorge Correia-Pinto, Ana Lobo

**Affiliations:** 1 Department of Pediatrics, Unidade Local de Saúde de Braga, Braga, PRT; 2 Department of Pediatrics, Unidade Local de Saúde do Alto Ave, Guimarães, PRT; 3 Department of Pediatric Surgery, Unidade Local de Saúde de Braga, Braga, PRT; 4 Life and Health Sciences Research Institute, School of Medicine, University of Minho, Braga, PRT

**Keywords:** bacterial pyomyositis, conservative management, iliopsoas abscess, pediatric hip pain, pediatric infection

## Abstract

Hip pain in children and adolescents poses a diagnostic challenge due to various underlying causes, ranging from benign to severe conditions. Presented here is the case of an otherwise healthy 14-year-old boy who arrived at the emergency department with a two-day history of left hip pain, limping, fever, anorexia, and vomiting. Upon physical examination, tenderness was noted upon palpation of the left sacroiliac joint and with mobilization of the left lower limb. Elevated inflammatory markers raised suspicion of an osteoarticular infection. Despite initially normal imaging, subsequent MRI revealed an iliopsoas abscess, later confirmed by blood cultures positive for *Staphylococcus aureus*. A conservative management with antibiotics was selected due to the infeasibility of percutaneous or surgical drainage, leading to clinical improvement. This case highlights the efficacy of non-surgical approaches in selected situations and underscores the importance of a thorough evaluation, early suspicion of infection, appropriate imaging, and tailored antimicrobial therapy in managing hip pain in pediatric patients.

## Introduction

Hip pain in children and adolescents is common but represents a significant diagnostic challenge, entailing a wide spectrum of causes, ranging from benign conditions such as transient synovitis and musculoskeletal injuries to more severe pathologies, including osteoarticular infections, inflammatory disorders, and malignancies [1-5]. A detailed evaluation is essential to distinguish among infectious, inflammatory, traumatic, and neoplastic etiologies [1-2]. Prompt diagnosis and appropriate management are crucial to prevent potential complications and ensure favorable outcomes.

Infection should always be considered in patients presenting with hip pain, especially when accompanied by fever, elevated inflammatory markers, and a history of rapid symptom progression [4,5]. Common bacterial infections, particularly involving *Staphylococcus aureus*, are well-documented causes of osteoarticular infections in pediatric populations [5]. However, less common etiologies, such as iliopsoas abscess, may also present with similar clinical features but are often not initially suspected, leading to diagnostic delays [6,7].

An iliopsoas abscess is a rare cause of hip pain in children, characterized by the collection of pus within the iliopsoas muscle [6]. Primary abscesses are relatively uncommon, particularly in the absence of a clear predisposing factor. This makes diagnosis challenging, as symptoms can overlap with more common conditions like septic arthritis, and initial imaging modalities may not detect the deep-seated infection [6,7].

This article was previously presented as an E-Poster at the Ninth Congress of the European Academy of Paediatric Societies in October 2022.

## Case presentation

**Figure 1:**
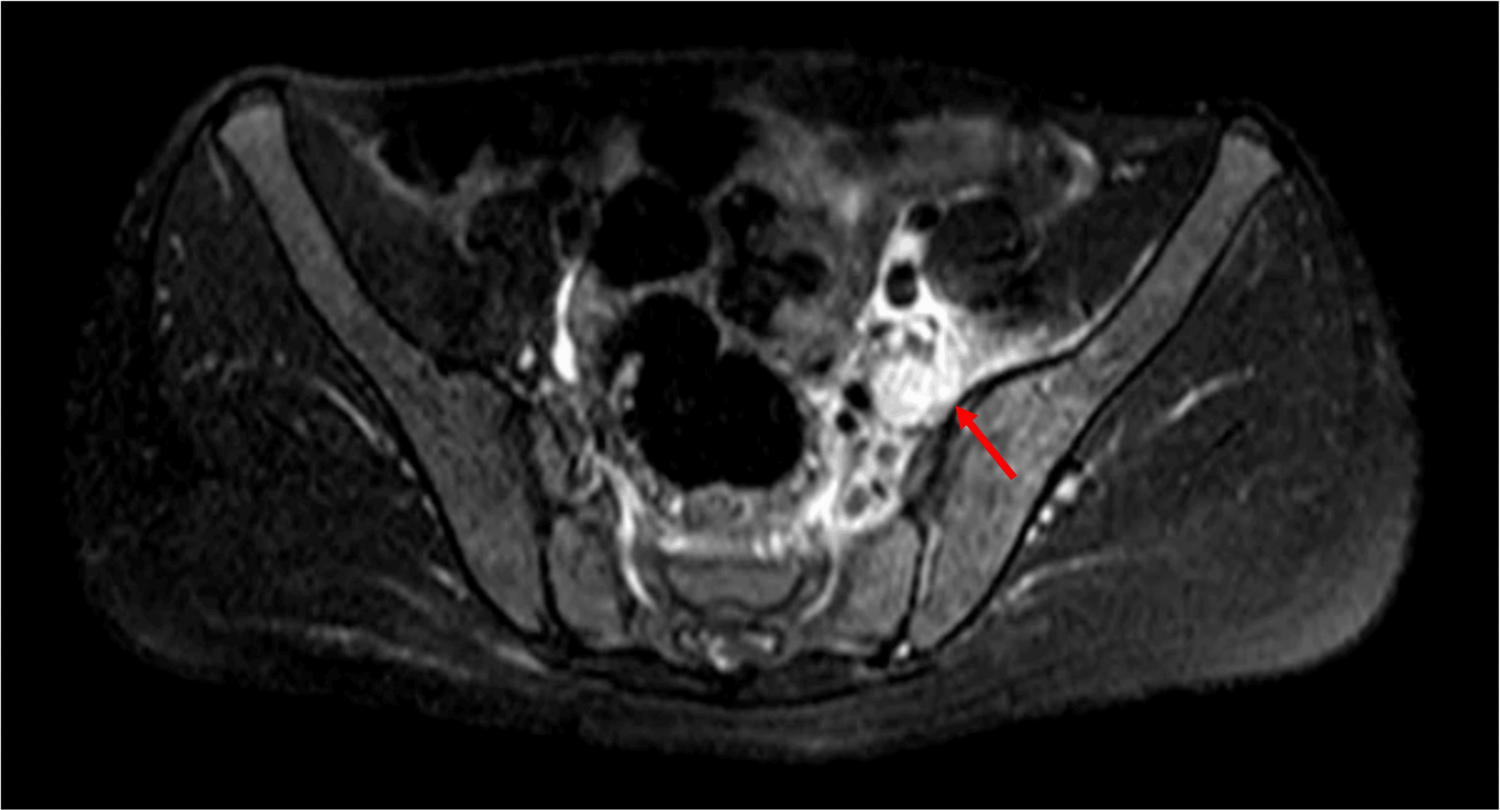
Pelvic MRI As seen in the image, marked by the red arrow, there is moderate edema of the soft tissues located between the left iliopsoas muscle, the iliac bone, and the lumbar spine, accompanied by moderate edema of the iliacus muscle.

An otherwise healthy 14-year-old boy presented to the emergency department with a two-day history of left hip pain radiating to the ipsilateral lower limb and lumbar region, with sudden onset and progressive worsening. The pain, described as mechanical, was circumscribed to the left sacroiliac joint. Upon admission, the patient exhibited fever, anorexia, and vomiting. The patient denied nocturnal pain awakenings, local inflammatory signs, or weight loss. No respiratory or urinary complaints were reported. There was no history of recent infection, trauma, or intense physical activity. Physical examination revealed discomfort upon deep abdominal palpation, an antalgic posture with hip flexion and adduction, and pain upon palpation of the left sacroiliac joint and gluteal region, as well as with limb mobilization. No deformities or inflammation was observed. No pain was noted on palpation of the spinous apophysis, paravertebral muscles, or other muscle masses. Laboratory findings indicated elevated C-reactive protein (CRP) (276.5 mg/L) and erythrocyte sedimentation rate (ESR) (58 mm/hour). Radiography and computed tomography (CT) of the hips were reported as unremarkable. Given the suspicion of osteoarticular infection, the patient was admitted for intravenous flucloxacillin (2 g every six hours). Blood cultures later identified *Staphylococcus aureus*. A subsequent MRI scan revealed an abscess within the iliopsoas muscles (Figure 1), which was later observed in a contrast-enhanced CT scan, measuring 74 × 47 × 21 mm. From day 4, the patient became apyretic and showed progressive clinical and analytical improvement. The Pediatric Surgery team from the reference hospital was consulted and the patient was subsequently transferred there, where a decision was made to adopt a conservative approach with ongoing laboratory and imaging reassessment. On day 10, there was evidence of laboratory improvement, with CRP of 49.6 mg/L and ESR of 59 mm/hour, and blood cultures were negative. However, a 72 × 27 × 17 mm abscess of the left iliac psoas muscle persisted on CT. Because of its location, with a high risk of lesions of major vessels, percutaneous or surgical drainage was not feasible. Conservative management was then maintained. By day 24, continued clinical, laboratory, and radiological improvements were noted, including a residual 5 mm collection on ultrasound. The patient was discharged on oral flucloxacillin, completing a five-week course of antibiotherapy. At one-month follow-up consultation, he remained asymptomatic. Immunodeficiencies tests were negative.

## Discussion

Acute severe hip pain, particularly when accompanied by fever, often indicates an infectious process [2,3]. Elevated white blood cell (WBC) count, ESR, and CRP levels can support this diagnosis [2]. In this case, normal initial imaging delayed the identification of a deep-seated iliopsoas abscess, later revealed by MRI and supported by blood culture results. 

Iliopsoas abscess is characterized by the accumulation of pus within the iliopsoas muscle [6]. It can be classified as primary or secondary, depending on its origin. Primary abscesses arise from hematogenous spread, while secondary ones result from contiguous spread from nearby infections [7,8]. Given the absence of an identifiable local source of infection in the presented case, the abscess is probably of primary origin, where the cause is typically unclear or idiopathic.

Clinical presentations of psoas abscesses may include symptoms like hip pain, back pain, limping, fever, and vague abdominal pain [6,7]. The patient referenced in this article presented with an acute onset of hip pain, limping, inability to bear weight, and fever, which prompted the provisional diagnosis of osteoarticular infection. Differential diagnosis between septic arthritis and psoas abscess can be challenging due to overlapping symptoms, but abdominal pain may lean more towards a psoas abscess [8]. Additionally, an antalgic position of hip flexion is characteristic during physical examination [6,7]. 

Standard laboratory investigations, including full blood count, CPR, and ESR, are useful in confirming the diagnosis of an inflammatory mass. Imaging findings are crucial in guiding the diagnosis and management strategy [7]. For the presented case, while initial radiographs and CT were unremarkable, MRI was key in the diagnosis and revealed an abscess. Plain radiography was the first imaging method, as it is recommended in the management of musculoskeletal pain to exclude fractures and changes in bone and joints [3-5]. Although ultrasound could have been considered for its non-invasive nature and soft-tissue imaging capabilities [3,6,9], it may not reliably detect early stages of pyomyositis [10]. If the cause of the pain remains elusive, axial imaging should be carried out [2]. CT can be normal in the early stages of pyomyositis since it is not sufficiently sensitive to evaluate soft tissues [7,11]. In fact, MRI is considered to be the best examination for early diagnosis of soft-tissue disorders, delineating the abscess wall and adjacent structures [4,7,9,11]. 

The isolation of *Staphylococcus aureus* from blood cultures further confirmed the infectious nature of the condition, underscoring the role of bacterial infections in acute hip pain and the necessity for prompt antimicrobial therapy. Culture typically yields positive results, with *Staphylococcus aureus* being the most common causative agent [7,9,11], as seen in this patient.

Treatment options for psoas abscesses include antibiotic therapy, drainage, and debridement [7]. Empiric antimicrobial therapy with coverage of *Staphylococcus aureus* is the first-line approach [6,7]. Percutaneous radiologically guided drainage stands as an effective and safe alternative to open surgical drainage. However, in cases where less invasive methods fail or when the abscess is secondary to intra-abdominal pathology requiring surgical intervention, open drainage becomes necessary [7]. In the presented scenario, conservative management with flucloxacillin for five weeks led to substantial clinical and analytical improvement, indicating the efficacy of conservative approaches in selected cases, especially when surgical intervention poses significant risks.

## Conclusions

Iliopsoas abscess is an uncommon cause of hip pain, and diagnosis is often not considered at first, leading to delays in diagnosis and treatment. This case underscores the importance of a thorough clinical evaluation in children and adolescents presenting with hip pain. Early suspicion of infection, prudent use of imaging, and tailored antimicrobial therapy are key components of successful outcomes. Furthermore, this case contributes to the growing literature on conservative management of iliopsoas abscess, providing valuable insights for clinicians facing similar diagnostic and therapeutic dilemmas.
